# Supplementary material for: Hypoalbuminemia and Pharmacokinetics: When the Misunderstanding of a Fundamental Concept Leads to Repeated Errors over Decades
Source: Antibiotics (Basel). 2023 Mar 4;12(3):515. doi: 10.3390/antibiotics12030515 (PMC10044130; doi:10.3390/antibiotics12030515)
Supplement: Supplementary file 1 [file antibiotics-12-00515-s001.zip › antibiotics-2182213-Supplementary material-070323.pdf]

antibiotics

Supplementary material

Hypoalbuminemia and Pharmacokinetics: when the Misunderstanding of a Fundamental Concept leads to Repeated Errors over Decades

P. Gandia [1,2]*, S. Decheiver [1], M. Picard [1], R. Guilhaumou[3], S. Baklouti [1,2], D. Concordet [2]

Supplemental movie S1: Using a dynamic approach, this sound video covers various events from the initiation of drug perfusion to a patient without hypoalbuminaemia who suddenly presents severe hypoalbuminaemia.

Black dots represent free albumin while **green** dots mimic drug binding. **Red** dots correspond to the drug circulating into the body. The various colors are postponed in the graphical representation of those phenomenon with concentrations on y axis and divers' stages on x axis.

Supplemental table S1: The list of screened articles and an analysis of their contents (regarding the interpretation of hypoalbuminaemia on unbound/free exposure).

Selected articles have been classified in three divers' categories in terms of **accepted**, **ambiguous**, or **wrong** assertion regarding free fraction of various drugs.

Table S1 (Appendix A): Review of approved explanations regarding unbound fraction. Items in green are accepted.

Right explanations	Page	Year	References
"The finding that fub for minocycline was constant is consistent with the current understanding of the rapid equilibrium assumption between protein-bound and unbound drug. In short, **the protein-bound fraction of drug appears to act as a reservoir within the central compartment as it is expected that only unbound drug is distributed to tissues and/or eliminated from the body. As unbound drug distributes to tissues and/or is eliminated from the body, bound drug rapidly dissociates from albumin and other circulating proteins**. This rapid dissociation preserves the equilibration between bound and unbound drug and results in proportional distribution and clearance terms between the bound and unbound fraction of drugs."	10	2021	[1]
"**The pharmacokinetics of daptomycin should probably be evaluated based on its unbound concentration, particularly in patients with hypoalbuminemia due to critical illness, aging, chronic kidney disease (CKD), and liver cirrhosis**."	1042	2021	[2]
"**A previous study indicated that although clearance of total daptomycin (CL) was affected by alterations in fu, CLu did not get affected**. In our study, fu ranged from 0.05 to 0.14 depending on the influence of serum albumin, BUN, and FBG. Regarding factors affecting fu and CL, reports on teicoplanin, which is an antimicrobial agent with a high protein binding rate, identified serum albumin and FBG. In our study, the results were similar to those of teicoplanin, and, to the best of our knowledge, this is the first report on factors that affected fu in daptomycin. **Moreover, our results suggested that because CL varies with the influence of fu, establishing the dose using total concentrations may result in an under- or overestimation**."	1047		
"**We demonstrated that the optimal PK model using unbound daptomycin concentrations in nonobese elderly patients with hypoalbuminemia and CKD featured adding eGFRcys and age as covariates of CLu. In addition, our study suggested that the use of total concentrations may result in under- or overestimation due to alterations in fu**. Optimal daptomycin doses differed depending on the distribution of eGFRcys and age, and the standard dose may be insufficient for some patients. From these results, it is necessary to select daptomycin doses based on age and eGFRcys in nonobese elderly patients with hypoalbuminemia and CKD."	1054		
"Ceftriaxone protein binding was best described using a model with non-linear protein binding and one saturable binding spot. B_{max} was highly variable and dependent on serum albumin concentration. The model developed in this study is similar to models of ceftriaxone plasma protein binding in severely ill and healthy individuals. **Hypoalbuminaemia could lead to elevation of the unbound fraction, which is compensated for by an increase in clearance**. This was confirmed by the results of the simulations in the present study, indicating that **serum albumin concentrations need not be considered relevant for dosing adjustments**."	1557	2020	[3]
"In addition, given the extensive binding of posaconazole (99%) to plasma proteins, we hypothesised that **altered protein binding may occur in patients with altered albumin concentration, with variable effects on total and unbound posaconazole exposure**."	2	2019	[4]

"Generally, for both prophylaxis and treatment, an increase in BMI required an increased dose to achieve target total and unbound trough concentrations, whereas for a specific BMI value, a **decrease in albumin concentration did not alter the dosing requirements when considering the unbound trough concentration target but increased the dosing requirement when considering the total trough concentration target**."	6	2019	[4]
"Put altogether, **dosing prediction using total concentration targets would result in unnecessarily high doses in patients with hypoalbuminemia** and progressively lower doses with increasing albumin concentration, which are sub-optimal, when compared to predictions with the unbound target. The clinical relevance of these results is that **unbound but not total trough concentration targets should be used to determine dosing regimens of posaconazole or performing therapeutic drug monitoring guided dose adjustment when warranted**. Ideally, clinicians should rely on unbound concentration monitoring particularly in patients with marked hypoalbuminemia."	7		
"The effect of **changes in the free fraction of any drug are well known: a lower total concentration, a higher apparent CL, and an unchanged concentration of unbound, active drug**. An increased awareness of the effect of albumin concentrations would eventually result in a better interpretation of PK and PD changes."	283	2018	[5]
"Micafungin is highly protein bound in plasma (99.8%), mainly to albumin and α1-acid glycoprotein, which is concentration-independent over a range of 10-100 mg/L. [...] The effect of hepatic impairment was investigated as part of the registration studies in eight volunteers with moderate hepatic impairment due to hepatitis C, primary biliary cirrhosis, or alcohol abuse, with Child-Pugh scores ranging between 7 and 9. Exposure after a single 100 mg dose was decreased to a mean of 98 mg h/L, versus 126 mg h/L in matched healthy volunteers. [...] A possible explanation can be found in **decreased levels of albumin, resulting in an increased free fraction of micafungin. This results in a lower total plasma concentration and explains the decrease in AUC. Nevertheless, this decreased AUC is not considered to be clinically relevant, and no dose adjustments are recommended** for patients with moderate or severe hepatic dysfunction."	272		
"Hypoalbuminaemia in the critically ill is common, and a previous study demonstrated poor predictive performance of calculated unbound drug concentration using conventional method. **Given the variability of unbound drug fraction in the setting of hypoalbuminaemia, calculation of the unbound b-lactam antibiotics concentration using published protein binding data tends to underestimate the true unbound concentration of highly protein bound b-lactams, while these overestimate the unbound concentrations of some important b-lactams such as meropenem and piperacillin/tazobactam**."	3090	2018	[6]
"**In the setting of hypoalbuminaemia in the critically ill, there is a risk of underestimating unbound antibiotic concentrations when correcting total measured concentrations with published protein binding data. This can lead to inappropriate overexposure of drug and potential dose-related adverse events**."	3091		

"In an often-cited paper, Benet and Hoener argue convincingly that – with rare exceptions – **'changes in plasma protein binding have little clinical relevance'**, and cite ceftriaxone as one of the drugs 'for which changes in protein binding have been [erroneously] thought to be important'. In short, initially higher unbound concentrations due to an acute elevation of the unbound fraction are **rapidly counteracted by the higher amount of drug cleared from the plasma**. In the end, the **exposure to unbound drug (in terms of area under the curve) depends only on the clearance of unbound drug, which depends e.g. on renal function but not on the unbound fraction**."	530	2015	[7]
"To summarize, we used ultrafiltration to determine the unbound concentrations of ceftriaxone in plasma, and to describe the protein-binding characteristics and the plasma pharmacokinetics of ceftriaxone in intensive care unit patients. In accordance with previous studies, the volume of distribution and the unbound fraction were elevated, the latter particularly in patients with renal impairment or severe hyperbilirubinaemia. **Clearance of unbound ceftriaxone as the dominant pharmacokinetic parameter was proportional to renal function**. In patients with normal or reduced renal function, the standard dose (2 g once daily) resulted in sufficient unbound plasma concentrations above the susceptibility breakpoint throughout the whole dosing interval."	532	2015	[7]
"WHAT THIS STUDY ADDS: **Protein binding of ceftriaxone is reduced in intensive care unit (ICU) patients, not only because of hypoalbuminaemia. This does not impair the attainment of the pharmacokinetic (PK)/pharmacodynamic(PD) target *ƒ*T>MIC**. Plasma albumin concentrations and in vitro binding data from healthy volunteers cannot be used to predict unbound concentrations of ceftriaxone correctly in ICU patients."	525		
"Wong et al. have compared the measured free concentration (using ultrafiltration) with the free concentration predicted from published protein binding values for seven β-lactam antibiotics using blood samples obtained from critically ill patients. Significant differences between measured and predicted free drug concentrations were found only for highly protein-bound β-lactam antibiotics such as flucloxacillin (bias of 56.8% overprediction) and ceftriaxone (bias of 83.3% overprediction). **No correlation between free and bound concentrations was found for these antibiotics, therefore direct measurement is considered essential for these drugs. For low to moderately protein bound antibiotics (such as piperacillin and meropenem), free concentrations appear to be predictable from the total concentrations**."	6	2015	[8]
"**To the best of our knowledge, this study is the first demonstration that patients with hyperglycaemic hypoalbuminaemia display lower serum total TEIC concentrations and decreased TEIC association constants for albumin**."	166	2015	[9]
"In clinical samples from ICU patients, fu was independent of total concentration of vancomycin. **We could not demonstrate a significant correlation between fu and the albumin concentration in clinical samples. This is surprising, since vancomycin is believed to bind mostly to albumin,** and since our own in vitro data and experiments by others demonstrated a clear relationship. The dependency of binding on protein concentration and the independency from total drug concentration is a typical property of relatively weakly bound drugs with dissociation constants much larger than therapeutical drug concentrations."	322	2014	[10]

"**However therapeutic drug monitoring of free concentrations of antiretroviral drugs are at this point in the preliminary stage and more studies are needed with larger patient base as well as clinical correlations in order to establish guideline for monitoring free drug concentration of antiretroviral drugs**."	5	2007	[11]

Table S1 (Appendix B): Review of ambiguous explanations regarding unbound fraction. Items in **green** are accepted, items in **orange** are ambiguous and items in **red** are wrong.

Ambigous explanations	Page	Remarks/CASE	Year	References
"We found that protein binding of flucloxacillin is saturable in the therapeutic concentration range, which is in line with previous findings. **Furthermore, the unbound fraction increased with lower serum albumin. This is especially relevant for critically ill patients,** with reported incidences of hypoalbuminaemia as high as 40%-50%."	3225	Case B	2021	[12]
"We showed that the highly variable unbound fraction of teicoplanin could not be predicted using albumin levels. Because of the relatively high inter-individual variation in unbound teicoplanin concentrations that cannot be predicted with covariates, **routine therapeutic drug monitoring of unbound concentrations may be recommended in the clinic to guide treatment optimization in critically ill pediatric patients**."	10	Case D	2021	[13]
"**Nonlinear saturable protein binding best described the relationship between CEFt and CEFu. Albumin concentration is the only covariate that explains a significant part of the variability in protein binding in the present study.** This confirms ceftriaxone protein binding relationships described in previous PK studies."	7	**Ambiguous sentence:** This sentence wrongly suggests to the reader that protein binding can influence the unbound/free concentration.	2021	[14]
"Hypoalbuminemia always develops during CPB, and hypotension and hypothermia may also develop during the procedure. Cefazolin may be particularly susceptible to these physiological changes because it has a small distribution volume (11 L) and exhibits extensive and saturable binding to plasma albumin. **We previously reported that the mean plasma unbound fraction of cefazolin during CPB was increased by twofold compared with that before CPB**."	736	Case C	2021	[15]
"**The significant increase in the plasma unbound fraction of cefazolin during CPB was considered to be associated with hypoalbuminemia due to the expanded volume of distribution (V plus VCPB), as shown in the present study, in addition to saturable plasma protein binding of the drug reported previously.** Collectively, our final PPK model incorporating CPB as a covariate describes well the plasma total and unbound cefazolin concentrations measured in patients undergoing cardiac surgery with CPB."	742	Case E	2021	[15]

"In previous studies, plasma concentrations of cefazolin were measured in adult patients undergoing cardiac surgery with CPB. However, the authors measured only total plasma concentrations of the drug, and they estimated unbound drug concentrations by multiplying an unbound drug fraction reported in healthy subjects (i.e., 0.8). **In the present study, we revealed that the plasma unbound fraction of cefazolin is increased by more than twofold (i.e., 0.4) during CPB. Our study suggested that direct measurement of unbound cefazolin concentrations is essential for making a PPK model to predict a dosing regimen of the drug**."	742	Case C + **Ambiguous sentence:** Even though the conclusion is correct, the argument used is false. It's not because the free/unbound fraction increases that the unbound/free concentration increases too. The direct measurement of unbound/free concentration is needed because hypoalbuminemia can lead to misinterpretation of total concentration values.	2021	[15]
"**Lower albumin concentrations may lead to variations in the unbound fraction (*f*u) that may consequently affect other PK parameters**, as only the *f*u can distribute to the peripheral tissues, be filtered by the glomerulus, and, in the case of CVVHDF, be eliminated through the filter pores."	1170	Case C + D	2021	[16]
"Probably due to this dramatic increase in the *f*u, our patients exhibit a higher ceftriaxone CL than healthy volunteers or critically ill patients with sepsis, septic shock, and different degrees of renal function, a CL that is dependent on albumin concentration and weight based on the results of the population PK model. **Surprisingly, in spite of the augmented CL, dosing simulations show that, for an MIC ≤2 mg/L** (the clinical breakpoint for susceptibility to ceftriaxone), **a dose of 1000 mg q24h maintains unbound ceftriaxone concentrations for a 100% of the dosing interval above the MIC regardless of albumin concentration and body weight**."	1173	Case D		
"**Hence, the total flucloxacillin plasma concentrations poorly reflect the unbound concentrations.** The extent of plasma protein binding is highly relevant, because the **unbound fraction of the drug is responsible for its pharmacological effect**."	1845	Case B	2021	[17]
"**Critically ill patients with MSSA-BSI may be at risk for flucloxacillin overdosing as a consequence of impaired renal function and low serum albumin levels**."	1851	Case A		

"**Calculation of the unbound concentrations, assuming 95% protein binding, may therefore result in considerable overdosing, in particular in critically ill patients with hypoalbuminaemia and renal impairment.** In the present study, the inter-individual unbound plasma fraction of flucloxacillin varied widely from 1.1% to 64.7%, showing a substantially higher median value (11%) than reported for healthy individuals (5%)."	1852			
"Disease severity is likely associated with a catabolic condition and hypoalbuminaemia, and renal function is associated with the renal elimination proportion of flucloxacillin (Q0 = 0.3). **Similarly, unbound flucloxacillin concentrations and unbound fractions were substantially higher in critically ill patients than in those who were non-critically ill.**"	1852	Case A<br>+<br>**Ambiguous sentence:**<br>Because we don't know if they have measured or estimated the unbound/free concentration, readers can wrongly interpret that a variation of unbound/free fraction is associated to a variation of unbound/free concentration.	2021	[17]
"**Because critical illness affects albumin concentrations, which can change throughout hospitalization, and β-lactam efficacy is dependent on free (non-protein-bound) concentrations, we investigated the percentage of drug bound to protein.** We found that the percentage of bound β-lactam antibiotic had high inter- and intrapatient variability."	568	**Ambiguous sentence:**<br>This sentence wrongly suggests that protein binding influences unbound/free concentration.	2021	[18]
"SAFE Study Investigator reported that approximately 40% of critically ill patients presented with hypoalbuminemia, because, meropenem, and imipenem are highly bound to albumin, which could **increase the unbound fraction significantly**. **Therefore, various pathological characteristics in critically ill patients may induce a wide discrepancy in the unbound fraction concentrations.**"	587	Case C	2020	[19]
"Our study has several limitations. […] However, **hypoalbuminaemia mostly affects the highly protein-bound b-lactams,** where the number of samples is minimal in this cohort."	5	**Ambiguous sentence:**<br>This sentence is confusing as it suggests to the reader that the unbound/free concentration is changed.	2020	[20]
"Various studies have shown that **pathophysiological changes related to critical illness (i.e., altered fluid status, changes in serum albumin concentrations, renal and hepatic dysfunction, systemic inflammatory response syndrome, and microvascular failure) substantially change the pharmacokinetics (PK) and thereby the exposure to antibiotics.**"	2	Case A + E	2020	[21]

"It is an intuitive belief that an increase in f_u implies an increase in the exposure to unbound concentrations, leading to an increase in drug effect. However, from theoretical PK principles, CLu is independent of fu in most of the cases, whereas the clearance of total concentration depends on f_u: CL x CL_u. This principle was verified for daptomycin, since AUC_u was independent of protein binding, whereas AUC increased with protein binding. For instance, AUC was approximately halved for a patient with an f_u of 12% compared to a patient with an f_u of 7%, whereas AUC_u was unchanged. This leads to the important, and maybe counterintuitive, consequence that a modification of protein binding has no significant impact on daptomycin effect. However, when the PK parameters are calculated for total concentrations (e.g., AUC), results should be interpreted by keeping in mind that they depend on protein binding. For instance, a patient with an AUC_u that does not necessitate dosage adjustment, but with a high f_u (e.g., because of a low alb or any other reason), will have a low AUC. In this case, the AUC value would falsely suggest that the patient is underdosed."	7	Case D	2019	[22]
"In addition, low serum albumin concentration is frequently observed in ICU patients, leading to an increase in the free fraction of the beta-lactams highly bound to plasma proteins, such as cefazoline, ceftriaxone, or ertapenem. Thus, hypoalbuminemia may lead to increased Vd and tissue penetration, and also increased elimination, of beta-lactam antibiotics by glomerular filtration and/or metabolic clearance. This has been particularly observed for ceftriaxone or ertapenem."	4	Case E	2019	[23]
"The binding of beta-lactams to albumin and plasma proteins determines the free fraction, which is the biologically active fraction that diffuses across biological membranes to tissues. The free fraction is also the fraction that is eliminated by renal and liver clearance. When plasma protein amount decreases, the capacity of beta-lactams to bind to protein decreases and beta-lactam-free fraction increases. Previous studies have shown that the binding of beta-lactams to plasma proteins in ICU patients is highly variable and is more altered for antibiotics highly bound to plasma proteins in conditions of homeostasis (e.g., ceftriaxone, cefazolin, or ertapenem). As a result, plasma concentration of beta-lactam antibiotics may be lowered and more unpredictable in patients with severe hypoalbuminemia."	5	Case B		

"However, **the relationship between proteins and plasma-free concentration is not straightforward**. Firstly, a correlation between the free fraction and albuminemia has been shown for several beta-lactams such as flucloxacillin but is not proven for all beta-lactams. Secondly, **although the increase of its free fraction increases beta-lactam antibiotic clearance, it also increases its activity and this change may have little clinical consequence if unbound concentration remains almost unchanged.** As a result, total and free plasma concentration of beta-lactam antibiotics is unpredictable and measuring albumin (or at least plasma proteins) could provide valuable information on the expected pharmacokinetics variability. In addition, as most laboratories currently measure the total beta-lactam concentration, protein and/or albumin level is important to measure at the same time as beta-lactam concentration in order to interpret properly TDM results and decide whether the daily dose of beta-lactam requires adaptation, especially when an intra-patient concentration variability is observed."	5	**Ambiguous sentence:** The relation is easy to understand as hypoalbuminemia implies a decrease of the total concentration while the unbound/free concentration is unchanged. +<br>Case D		
"Third, **binding ratios reported for less sick patients or healthy individuals may not reflect those for critically ill patients because of the high variability in plasma protein concentration and altered binding properties in the critically ill. Therefore, the use of unbound pharmacokinetics in this study enables a more reliable prediction of optimal ceftolozane-tazobactam dosing**."	5	**Ambiguous sentence:** This sentence is confusing as it suggests to the reader that the unbound/free concentration is changed.	2019	[24]
"**Hypoalbuminemia, a common condition in critically ill patients, can also reduce plasma oncotic pressure leading to fluid extravasation and antimicrobial dilution**."	1	**Ambiguous sentence:** Even if the volume of distribution of the unbound/free form is increased due to fluid extravasation, this phenomenon has no consequence on the antimicrobial effect of the drug because the AUC is unchanged while the C_{min} is increased.	2019	[25]
"**A common occurrence in critically ill patients is the finding of increased renal clearance due to an increased cardiac output and an increased volume of distribution because of third spacing and hypoalbuminemia.** For betalactam antibiotics specifically, both findings may substantially lower antibiotic concentrations."	76	**Ambiguous sentence:** Hypoalbuminemia increases the total volume of distribution but not the unbound/free one.	2019	[26]
"Third, no unbound TZP or MER concentrations were measured. **Only the unbound fraction of the antimicrobial drug is able to exert its antibacterial effect.** Therefore, a level of 30% and 2% protein binding was assumed for TZP and MER respectively, which was previously seemed appropriate."	78	Case C		

"Since temocillin is highly bound to human serum proteins (80%), the **impact of protein binding on MIC was assessed *in vitro* by adding human serum or albumin to the medium. A two- to fourfold increase of MIC was observed in the presence of human serum, but the maximal killing rate (obtained at a concentration of four times the MIC) was not impaired by the addition of human serum, suggesting that the impact of protein binding on temocillin activity *in vivo* may be limited**."	4	**Ambiguous sentence:**<br>Transposing *in vitro* temocillin protein-binding on *in vivo* condition is an usual mistake leading to confusion for medical practice.<br>Indeed, what happens in *in vitro* (i.e., static) condition does not reflect what happens in *in vivo* (i.e., dynamic) one.	2018	[27]
"**Nonetheless, hypoalbuminemia associated with DKD may interfere more with free blood fraction of rifapentine than with that of rifampicin**."	2968	Case C	2017	[28]
"As protein binding was altered and highly variable, our study aimed to compare target attainment rates using different proposed PK/PD targets, i.e. total (trough) concentration and (f)AUC/MIC. [...] Since trough concentrations of 10–15 mg/L are believed to be a good surrogate to achieve an AUC/MIC 400, **it would be reasonable to assume that the latter target would not be achieved in the majority of patients.** [...] **In clinical practice, monitoring vancomycin exposure by fAUC (with MIC if available), and thereby taking into account the protein binding, might be a more justified target to prevent underdosing or overexposure.** Furthermore, given the high variability in protein binding in our study population, it seems not advisable to assume a fixed unbound fraction to calculate this fAUC/MIC ratio. **In this heterogeneous population with different types of infection, we were able to accurately predict the unbound vancomycin concentration in plasma based on total vancomycin and total protein concentration**."	803	**Ambiguous sentence:**<br>This sentence wrongly suggests that, while total exposure varies with hypoalbuminemia, the unbound/free one is also modified.<br><br>**Ambiguous sentence:**<br>Predicting unbound/free concentration is not reasonable if a mean, and not an individualized unbound/free fraction, is applied into the mathematical model. Individualized values do not only depend on Bmax (total protein level) but also on Kd of albumin binding. The best method consists in determining the unbound/free concentration directly.	2017	[29]
"The presence of **hypoalbuminaemia, like numerous other conditions that are commonly seen in the critically ill, may lead to altered plasma ceftriaxone concentrations**. In the absence of therapeutic drug monitoring, it can be difficult to prescribe drugs such as ceftriaxone with confidence for critically ill patients and know that dosing is adequate."	1	Case A	2016	[30]
"There is a **large interindividual variability in total and unbound ceftriaxone pharmacokinetics in this population, which may be driven by one or more different conditions including hyperbilirubinaemia, diabetes, hypoalbuminaemia and CLCr**."	5	Case A		
"However, it has been reported that the free fraction of TEIC is increased in patients with serum albumin levels of <3.0 g/dL. **Moreover, patients with hypoalbuminemia were demonstrated to have lower serum through concentrations of TEIC than healthy**	164	Case E<br>+<br>**Ambiguous sentence:**<br>There is an ambiguity. This sentence is true in terms of	2015	[9]

**volunteers. Thus, it is considered that an increased unbound TEIC fraction results in a greater volume of distribution (Vd) or increased clearance (CL) of the drug, which can lead to a reduced serum concentration of TEIC**."		total concentration/total pharmacokinetics but false if it refers to unbound/free one.		
"Likewise, **the results of the current study can be explained by the hypothesis that the conformational changes in the albumin molecule induced by glycosylation decrease its TEIC binding capacity, resulting in an increase in Vd**."	167	Case E		
"Moreover, a 4 g daily dose divided into two administrations at a 12 h interval **may not be sufficient in critically ill patients where alteration of critical parameters, such as drug volume of distribution (*V*), CL and protein binding, as well as end-organ dysfunctions, may markedly alter antibiotic disposition and potentially reduce the efficacy of anti-infective treatments and adversely affect patient outcome**."	892	Case A + E	2015	[31]
"To summarize, we demonstrated that determination of unbound plasma concentrations of ertapenem by ultrafiltration is susceptible to experimental conditions. **Mimicking physiological conditions, we found higher unbound concentrations in plasma from healthy volunteers than previously reported and these were even higher in samples from ICU patients, providing reasonable exposure to free drug with standard dosing. Our results suggest influences beyond hypoalbuminaemia in ICU patients**."	3110	Case A	2014	[32]
"Finally, **conflicting information has been published in the past about the clinical relevance of increased unbound drug concentrations in cases of hypoalbuminemia**. However, in these studies, the drugs that were the subject of debate were mostly drugs with linear pharmacokinetics. The influence of hypoalbuminemia on protein binding characteristics of antimicrobial agents was demonstrated in critically ill patients for highly protein-bound drugs (PPB above 70%) such as ceftriaxone (PPB, 95%), flucloxacilline (PPB, above 90%), carbamazepine (PPB, 70% to 80%), phenytoin (PPB, 90%), and valproic acid (PPB, 80% to 90%). For drugs with low to moderate PPB (30 to 70%) and linear pharmacokinetics, changes in PPB have little consequence in clinical practice, as small increases in unbound drug concentrations are immediately metabolized and eliminated. To the best of our knowledge, we are the first to show the influence of hypoalbuminemia on the PPB of voriconazole, a drug with a nonlinear PK profile due to a saturated metabolism combined with a narrow therapeutic range and moderate PPB. Recently, unbound voriconazole	6785	Case C	2014	[33]

concentrations were also investigated with ultrafiltration (UF) by Florent et al. **A correlation was seen between the unbound voriconazole fraction and albumin plasma concentrations below 25 g/liter.** Overall, no clear correlation with plasma albumin concentrations was measurable."				
"Since variations in protein binding and the prevalence of hypoalbuminemia among critically ill patients have been observed in other studies, there is increasing concern regarding the accuracy of this estimation, especially for highly protein-bound drugs in critically ill patients. **Since the time course of unbound beta-lactam concentrations is more relevant than the total concentration, direct measurement of the unbound fraction has been suggested to have potential advantage in antibiotic dose optimization for critically ill patients**. In this study, we utilized a rapid and inexpensive assay for measurement of unbound beta-lactam concentrations in clinical practice. The data presented again demonstrate severely altered PK of beta-lactams in critically ill patients."	6167	Case C	2013	[34]
"Specifically, **we report three cases where maintaining optimal beta-lactam plasma concentrations was particularly challenging, primarily owing to the nature of the infection, reduced plasma protein concentrations and variable renal function**."	164	**Ambiguous sentence:** This sentence wrongly suggests that hypoalbuminemia influences unbound/free concentration.	2013	[35]
"**Augmented renal clearance (ARC), or the enhanced renal elimination of circulating solute, is also not infrequent in critically ill patients**."	165	Case E + **Ambiguous sentence:** Total clearance is augmented if unbound fraction and/or GFR increase(s) while unbound clearance is augmented if GFR increases.		
"Subject 3 received daptomycin 6.9 mg/kg and achieved the lowest observed C_{max} in this study, 44.7 µg/mL. Closer inspection of this patient indicated that he had profound hypoalbuminemia (serum albumin ranged from 16 to 18 g/L during the study time interval) and a corresponding mean daptomycin free fraction of 28.2%. **This subject also had the largest calculated apparent steady-state volume of distribution, likely as a result of the higher free fraction. Because the free fraction represents the portion available to exert a therapeutic effect, the higher free fraction may be associated with sufficient free daptomycin concentrations despite the low (total) Cmax observed**."	22	Case B	2011	[36]

"Only free drug is active and the unbound state is a prerequisite for tissue distribution. One strength of our study is the measurement of free drug levels. **According to a recent paper, it is recommended that all PK/PD indices should be referenced to the unbound (free) fraction of the drug.** As only free drug is the active moiety and the MIC is measured in a plasma protein-free medium, these recommendations appear to be justifiable. In contrast, the optimal PK/PD values appointed by clinical studies, which were also used as target values in this report, were determined by using total serum concentrations. **To compare our results with other studies and with target PK/PD values that are based on total drug levels, despite its pitfalls we were forced to make adjustments for protein binding.**"	165	Case B<br><br>**Ambiguous sentence:**<br>This approach can lead to interpretation errors, even if information is expressed in terms of total concentration in publications.	2007	[37]
"One important reason for the different pharmacokinetic data is that critically ill patients often present with several peculiar pathophysiological or iatrogenic conditions, which may substantially affect distribution and/or elimination of antimicrobial drugs. This may involve an increased volume of distribution (V), e.g. as a result of edema, pleural effusion, ascites, or indwelling post-surgical drainage, or an enhanced CLR, e.g. as a result of burns, hyperdynamic conditions in septic shock or use of haemodynamically active drugs. **In our patients without third-space characteristics, we suppose the enhanced V and CL_R with resulting low C_{max} and $AUC_{0-\infty}$ values were mainly related to the decreased serum albumin concentrations (range 9.2–25.6 g/L), as a consequence of the fluid therapy with a positive daily fluid balance (mean ± SD, 1.8 ± 1.0L/day). The same results with reference to hypoalbuminaemia-related V and CL_R enhancements in critically ill patients were previously reported by *Joynt et al.* for treatment with ceftriaxone and by *Pea et al.* for teicoplanin therapy in a renal transplant patient with septic shock.**"	282	Case E	2006	[38]
"Teicoplanin is extensively bound to serum proteins, mainly albumin, the free fraction being 6–12% irrespective of serum concentration. **Teicoplanin protein binding is likely to be altered in ICU patients, either due to the decrease in albumin level and/or to interactions with other drugs sharing the same binding sites. Because only unbound drug may distribute within tissues, total serum concentration is not an ideal parameter for rational dosing of antibiotics, especially for those compounds with extensive protein binding.**"	776	**Ambiguous sentence:**<br>Because unbound/free fraction and unbound/free concentration are mentioned in the same paragraph, readers can wrongly interpret that a variation of teicoplanin fraction is associated to a variation of unbound/free concentration.	2006	[39]

"**However, because the unbound fraction of teicoplanin in these patients was much higher than expected, possibly due to a half decrease in albumin level in serum, the median unbound trough serum concentration of teicoplanin was equal to 4 µg/ml, which is above the value of 2 µg/ml corresponding to a target trough total serum concentration of 20 µg/ml,** with an unbound fraction of only 10%. Interestingly, median ELF and unbound serum concentrations of teicoplanin were close to each other."	778			
"**Hypoalbuminemia is thus expected to result in a higher free fraction of ceftriaxone, with possible consequences on its clearance and distribution.** Moreover, ceftriaxone protein binding is known to be partly restrictive, this binding may hinder or prevent drug distribution or elimination, so that its bactericidal effect is mostly attributed to the unbound concentration rather than the total concentration. **Hence, a modification of free concentration kinetics could have an impact on drug effectiveness**."	736	Case E<br>+<br>**Ambiguous sentence:**<br>Total clearance and total volume of distribution will be augmented not the unbound/free ones. Moreover, the last sentence suggests to the readers that variations of the unbound/free fraction induce variations of the unbound/free concentrations.	2000	[40]
"Quinidine is 70 to 95% bound to plasma protein, primarily to albumin but also to a number of other plasma constituents. Binding is reduced in patients with cirrhosis, partly because of hypoalbuminaemia, but is not influenced by renal insufficiency. **Clinical interpretation of total serum or plasma quinidine concentrations must be altered in patients with reduced or increased binding, since it is the unbound fraction which is pharmacologically active**."	150	Case C	1980	[41]

Table S1 (Appendix C): Review of false explanations regarding unbound fraction. Items in green are accepted, items in orange are ambiguous and items in red are wrong.

False explanations	Page	Remarks	Year	References
"Teicoplanin is a mixture of several isomorphic components, including five major compounds (A2-1 to 5) accounting for 95% of the total product, an hydrolysis product (A3-1), and four minor (RS-1 to 4) compounds. All main compounds are extensively protein bound (total teicoplanin protein binding [> 95%]) but show slightly variable affinity to albumin. **Because of this extensive protein binding, changes in plasma protein concentrations can influence teicoplanin efficacy**."	4		2021	[13]
"Teicoplanin is a highly protein-bound drug and is mainly bound to plasma albumin, of which concentrations may greatly vary within and between critically ill patients. In our patient population, we observed such variability ($C_{albumin}$: median 30.0 mg/L, range 18-46 mg/L). **It has been shown that albumin levels significantly affect the unbound teicoplanin concentrations in adult patients**. However, in our study, we could not identify such a relationship. As we observed a high non-predictable variability in unbound fractions (f_u: median 0.083, range 0.036-0.28), these observations support the measurement of unbound teicoplanin concentrations for optimization of treatment."	9			
"**Albumin concentrations in our patients were low which could explain the higher unbound fractions of cefazolin which we observed.** Although low albumin concentrations are common in critically ill patients, further albumin sequestration and dilution due to the ECMO-circuit could be possible, as proteins bind to artificial surfaces such as ECMO-tubing and membrane oxygenators. Regardless of modern coatings and PMP-oxygenators, ECMO-circuits still show accumulation of blood components over time which leads to *membrane fouling*. **At the same time our findings endorse previous study-results that showed, predicting unbound fraction by using albumin concentration is probably not accurate as similar albumin concentrations in our patients still led to highly variable plasma concentrations. That is why measuring the unbound fraction of cefazolin seems to be important** as only then the biologically active concentration is determined. **High total plasma concentrations with high protein binding, for example, might still lead to insufficient active (= unbound) cefazolin levels and would be unrecognized, if only the total concentration is measured.** On the other hand, excessive cefazolin concentrations enhance the risk for adverse effects while the benefit of unbound concentrations above 16 mg/L is debatable."	6	Case C	2021	[42]

“We recognise that our case series is limited and that the study design was retrospective and monocentric. Additionally, **only total cefiderocol concentrations were measured, thus potential variability in protein binding commonly encountered in critically ill patients could impact on cefiderocol free levels**.”	297		2021	[43]
“**The impact of ceftriaxone pharmacokinetic alterations on protein binding and PK/PD target attainment still remains unclear.** We evaluated pharmacokinetic/pharmacodynamic (PK/PD) target attainment of unbound ceftriaxone in critically ill patients with severe community-acquired pneumonia (CAP). Besides, we evaluated the accuracy of predicted vs. measured unbound ceftriaxone concentrations, and its impact on PK/PD target attainment.”	1		2021	[14]
“In the ICU setting, **where hypoalbuminemia is present in up to 50% of all patients, the unbound ceftriaxone fraction is highly variable.** This is probably due to its nonlinear concentration-dependent protein binding. Measuring the unbound fraction using equilibrium dialysis, which is the reference method, is time-consuming and costly. Therefore, there have been several attempts to estimate unbound ceftriaxone concentrations (CEFu), according to a fixed percentage of protein binding or to a predictive protein binding model. **Still, the impact of using protein binding models for ceftriaxone on PK/PD target attainment has not been investigated**.”	2	Case C		
“However, we hypothesized that in patients with septic shock, hypoalbuminemia, and CVVHDF requirement, **ceftriaxone *fu* would be increased, thus augmenting ceftriaxone CL and therefore compromising the attainment of therapeutic concentrations over the 100% of the dosing interval**.”	1175	Case E	2021	[16]
“Data has shown that ceftriaxone unbound concentrations in critically ill patients were > 40%, **much higher than in non-critically ill patients, because of hypoalbuminemia**.”	1179			
“**The volume of distribution of many drugs increases with body size, and circulating plasma proteins like albumin can directly contribute to reducing the free fraction of many drugs. While studies suggest that systemic exposures are reduced in patients with hypoalbuminemia due to increases in Vss and CL secondary to increased fub**, we did not find this to be the Case Eor minocycline, as CL was found to be independent of albumin.<br>We do not anticipate that BSA differences across critically ill patients will have any bearing on achieving critical PK-PD targets since changes in Vc do not affect the AUC. **However, the association between fub and albumin may have implications for clinical practice for critically ill patients**	12	Case C + E<br><br>**Wrong sentence:**<br>There is a confusion between the PK of total concentration and the PK of unbound/free	2021	[1]

**as the extent of unbound drug is driven, in part, by fub. Given the predicted increase in fub over the albumin range of 1 g/dl to 3.6 g/dl, the probability of PK-PD target attainment profile may be less robust among individuals with albumin levels in the normal range than among those with extremely low albumin concentrations.** However, the model predicts that a doubling in the fub will shift the probability of target attainment profile downward, at most, by only 1 MIC doubling dilution, assuming there are no corresponding changes in CL [...] However, the findings from ACUMIN should be applied only to critically ill patients with similarly low albumin concentrations. **Studies suggest that systemic exposures are reduced in patients with hypoalbuminemia due to increases in Vss and CL secondary to increased fub. Although we did observe an association between CL and albumin, the extent of protein binding observed in the ACUMIN study population may have contributed to the faster CL values reported in this study relative to historical values.**”		concentration that leads to wrong conclusions.		
“Therefore, high variability in free concentrations can affect clinical outcomes. **There are likely multiple factors contributing to the large variability in free concentrations found in our study, including pathophysiologic changes from critical illness and variability in protein binding, as well as patient factors. Because albumin is the primary protein that binds drugs, hypoalbuminemia, commonly seen in critical illness, may lead to an increase in the unbound fraction of the drug. However, hypoalbuminemia increases Vd, lowering free drug concentrations. Furthermore, because the unbound drug fraction is the portion cleared by the kidneys, an increase in free drug concentrations may lead to an increase in drug clearance, and thus result in overall low drug exposure.** Albumin concentration may change frequently based on clinical status, leading to intrapatient variability in antibiotic protein binding. Thus, it is important to evaluate total and free concentrations to ensure efficacy while limiting adverse events.”	570	Case C + E<br><br>**Wrong sentence**<br>This sentence wrongly suggests that unbound/free concentration influences drug clearance.	2021	[18]
“These pathophysiological changes, for example altered renal function or **hypoalbuminaemia**, can influence antibiotic pharmacokinetics (PK) and consequently the achievement of PK/**pharmacodynamic (PD) targets**.”	2641		2020	[44]
“The effect of hypoalbuminemia for **cefuroxime dosing in critically ill patients with low levels of albumin** or renal failure **is likely to have significant consequences on the drug's pharmacodynamics** and pharmacokinetics.”	2		2020	[45]

"Usually, several conditions are observed in critically ill patients, including impaired renal and liver outcomes and drugs demonstrating an abnormal proportion of binding to albumin; **any alterations in this binding may induce changes in the unbound antibiotic concentration with minor or even no effect on total antibiotic concentrations**. Therefore, concentrations at site-specific infections are better correlated with the unbound rather than the total plasma concentrations. In addition, unbound antibiotic concentration monitoring may be of importance for TDM in critically ill patients."	578		2020	[19]
"Another limitation of this study is the lack of free tazobactam concentration data. **Dynamic changes and concentration-dependent protein binding could impact tazobactam concentrations at the site of action. Critically ill patients are more susceptible to changes in protein binding due to the underlying disease state and drug interactions.** Further studies that quantify free tazobactam concentrations should be considered to adequately predict the dosing regimens that optimize the target attainment of interest."	8		2020	[46]
"In patients with hypoalbuminaemia, reduced plasma-oncotic pressure further augments fluid shifts, leading to increases in volume of distribution for some drugs. **Hypoalbuminemia also results in a substantial increase in the unbound plasma concentration, particularly for highly protein-bound antibiotics, which means that more drug distributes into the interstitial space, with the increased fluid shift thereby accelerating the expansion in volume of distribution**. However, although the influence of hypoalbuminemia has been described for highly protein-bound drugs, **is it less frequently reported with drugs that are protein bound at low levels**."	2		2019	[24]
"However, previous studies on critically ill and neonates have suggested that flucloxacillin protein binding may be highly variable and dependent on serum albumin concentrations. **As only the unbound concentration is pharmacologically active, it is pivotal that in vivo flucloxacillin serum protein binding has been fully characterized when developing improved dosing regimens;** thus far, this has not been performed."	311		2019	[47]
"**The unbound fraction increased when serum albumin decreased, and unbound concentration increased**. These findings suggest that albumin concentrations affect binding capacity and that protein binding is saturable in the therapeutic range of flucloxacillin concentration."	312			

"However, currently available TDM reports have not directly accounted for **altered protein binding, and the associated change in unbound β-lactam concentrations**, despite the high prevalence of hypoalbuminaemia causing altered protein binding in the critically ill. Given that efficacy is dependent on the unbound rather than total plasma concentration of antibiotic, knowledge of unbound concentrations when applying TDM should be considered essential."	3088	**Wrong sentence:** Hypoalbuminemia does not influence unbound/free concentration.	2018	[6]
"In routine clinical practice, trough concentrations are used as a 'surrogate' parameter to optimize vancomycin dosing regimens, because AUC/MIC calculations are labour- and cost-intensive. **Both targets are based on total drug concentrations, whereas only the 'unbound' or 'free' drug exerts a pharmacological effect.** A more direct fAUC/MIC target > 200 has been advocated as the PK/PD target assuming a fixed unbound vancomycin fraction of 50%. **However, critically ill children exhibit marked variability in plasma protein concentrations (with albumin concentration ranging between 15 and 54 g/L), which may alter the protein binding. To date, no studies have investigated the implications of altered protein binding on target attainment rates.**"	[801]	**Ambiguous sentence:** This sentence wrongly suggests that hypoalbuminemia influences unbound/free concentration.	2017	[29]
"Rifapentine is highly bound (~99%) to plasma proteins; hence, **coadministered drugs may compete for the same plasma protein-binding sites and affect the free drug concentration.** This may be the case with their concurrent use with sulfonylureas (protein binding >99%), glinides (protein binding >98%), or SGLT-2 inhibitors (protein binding >98%). **The pharmacokinetic and clinical consequences of potential protein-binding displacement remain to be elucidated.**"	2960		2017	[28]
"Clinical data on the relationship between the pharmacodynamic and pharmacokinetic properties of detemir and degludec, and the plasma level of albumin are very scarce. **However, due to their high-level protein-binding, potential interactions of detemir and degludec with rifapentine should be considered.**"	2961		2017	[28]
"Hypoalbuminaemia, defined as a serum albumin concentration < 25 g/l, is present in 40–50% of critically ill patients, and has two prominent effects on the PK of β-lactam antibiotics. Firstly**, it increases the concentration of unbound antibiotic, which in turn is available for distribution and renal clearance. Secondly, it increases the volume of distribution of β-lactam antibiotics by augmenting fluid shifts into the interstitial space. This is particularly relevant for highly protein bound β-lactam antibiotics such as flucloxacillin, ertapenem and ceftriaxone. While hypoalbuminaemia may temporarily result in higher concentrations of highly protein bound β-lactam antibiotics**, a **reduced fT>MIC will eventually result as a consequence of an increased dilution and drug clearance**."	3	**Ambiguous sentence:** For flucloxacillin, ertapenem and ceftriaxone, the increase of unbound concentration occurs on a very short duration. Nobody has been able to evaluate the actual duration (a few seconds ??, a few minutes ??). However, unbound concentration comes back to the baseline level while unbound	2016	[48]

<table>
<tr><td></td><td></td><td>fraction (fu) increases. This increase of fu leads to an increase of the clearance and of the volume of distribution for total concentration but not for unbound concentration.</td><td></td><td></td></tr>
<tr><td>“The impact of decreased serum albumin concentrations on free antibiotic concentrations in non-critically ill patients is poorly described. This study aimed to describe the pharmacokinetics of a high-dose regimen of teicoplanin, a highly protein-bound antibiotic, in non-critically ill patients with hypoalbuminaemia. […] This study confirms the significant impact of hypoalbuminaemia on free concentrations of teicoplanin in non-critically ill patients, similar to that in critically ill patients. Furthermore, the poor correlation with total teicoplanin concentration suggests that therapeutic drug monitoring of free concentrations should be used in these patients.”</td><td>1</td><td rowspan="2">Ambigous sentence: This sentence wrongly suggests to the reader that the unbound/free concentration of teicoplanin has to be monitored because its value is increased when hypoalbuminemia.</td><td rowspan="2">2015</td><td rowspan="2">[49]</td></tr>
<tr><td>“It has become increasingly clear that the free (unbound) antibiotic concentration is responsible for the pharmacological effect and a better understanding of this can enhance the accuracy of therapy and potentially improve clinical outcomes. This might be of particular relevance for highly protein-bound antibiotics such as teicoplanin (90-95% bound), especially in critically ill patients where hypoalbuminaemia is frequent and as a consequence the volume of distribution (V) and clearance (CL) of the unbound drug are increased. These pharmacokinetic (PK) changes could result in suboptimal teicoplanin exposures and may necessitate dose adjustments to ensure that therapeutic exposures are achieved. This highlights three important shortcomings of available data on the pharmacokinetics of unbound teicoplanin. First, unbound concentrations are often not measured either clinically or for academic reasons. Second, in the rare circumstances that unbound concentrations are determined, patients generally have not had serum albumin concentrations that were sufficiently low to alter the free teicoplanin pharmacokinetics significantly. Third, no PK data are available describing the effect of hypoalbuminaemia on unbound concentration when using dosing regimens for non-critically ill patients with ‘deep-seated’ infections such as those of bone and prostheses, where the target total trough concentration (tCmin) is ≥ 20 mg/L […]. Data from the critical care literature suggest that hypoalbuminaemia is likely to alter teicoplanin pharmacokinetics significantly and, in particular, free concentrations.”</td><td>2</td></tr>
</table>

"We found that patients with hyperglycaemic hypoalbuminaemia had lower serum TEIC concentrations after a loading dose than patients with non-hyperglycaemic hypoalbuminaemia, hyperglycaemic normoalbuminaemia and non-hyperglycaemic normoalbuminaemia. However, in the hyperglycaemic hypoalbuminaemia patients, the serum trough concentration 12 h after a loading dose exceeded 10 g/mL when the loading dose was >1600 mg (data not shown). **Therefore, TEIC regimens with a high loading dose might be required for patients with hyperglycaemic hypoalbuminaemia to avoid low serum TEIC concentrations.** **In conclusion, patients with hyperglycaemic hypoalbuminaemia exhibit lower serum TEIC concentrations following administration of a loading dose.** In addition, it was suggested that glycosylated albumin decreases the association constant of TEIC for albumin. **A PPK study that includes glycosylation of albumin as a parameter is needed to identify the impact of albumin glycosylation on the pharmacokinetics of TEIC.**"	168	**Ambiguous sentence** There is a confusion between the total pharmacokinetics and the unbound/free ones. No TEIC dosage adjustment is required as unbound/free drug exposure is unchanged by hypoalbuminemia or glycosylation of albumin.	2015	[9]
"Dramatically decreased serum albumin concentrations are also common in these patients. **For a renally cleared antibiotic like cefazolin, which has high protein binding (90%), these changes can severely alter plasma concentrations.** **The effects of these changes on cefazolin concentrations in the interstitial fluid (ISF) of subcutaneous tissues, which is the site of infection for serious skin and soft tissue injury, are also unknown, but crucial to successful prescription.**"	1496	**Ambiguous sentence:** This sentence wrongly suggests that hypoalbuminemia influences unbound/free concentration.	2015	[50]
"This analysis has demonstrated that increasing CLCR or **decreasing serum albumin concentrations reduce the likelihood of achieving optimal cefazolin exposures in ISF**. [...] Cefazolin is a widely used prophylaxis and treatment option for severe wound infections in post-trauma critically ill patients. **It is highly bound to albumin (90%), suggesting that in critical illness and/or the presence of hypoalbuminaemia, altered pharmacokinetics are likely.** Such effects are likely to be common in critically ill patients, given that 40% will have serum albumin concentrations <25 g/L. **The increased unbound plasma concentration that results from such pathophysiological changes should lead to higher pharmacologically active concentrations** **but also capacity for increased drug clearance.** The simulations shown in Figure 5 support the contention that a higher albumin concentration results in better cefazolin exposures in ISF. **Without the reservoir of cefazolin bound to albumin to supplement unbound drug that is cleared from the body, patients with lower albumin concentrations are more likely to have reduced pharmacokinetic/pharmacodynamic target attainment.**"	1498	Case E		

"**A consequence of the higher unbound concentration in plasma is that it is available for renal elimination from the body and increased drug clearance is likely to result in increased CLCR.** Indeed, CLCR was a strong covariate in our model, which is in keeping with its renal elimination characteristics."	1500		2015	[50]
"An important characteristic of teicoplanin is its high protein binding (90%), which can lead to increased pharmacokinetic (PK) variability. Yano et al. demonstrated that plasma albumin concentrations are an important determinant in this variability, with lower albumin concentrations associated with higher unbound fractions of teicoplanin. **In critically ill patients, hypoalbuminaemia is a frequent phenomenon** and as such teicoplanin PK variability may be significant. **As the unbound or free concentrations are responsible for pharmacological activity, one could theoretically expect higher active concentrations of teicoplanin in these patients.** Furthermore, in critically ill patients, renal impairment frequently accompanies hypoalbuminaemia. **As clearance of the unbound drug occurs almost exclusively by glomerular filtration, the total body clearance of teicoplanin will decrease with increased renal impairment and will increase with decreases in protein binding. Augmented renal clearance is also common in critically ill patients, meaning that some patients may develop very low concentrations of renally cleared drugs such as teicoplanin. Consequently, free and total plasma concentrations are difficult to predict in critically ill patients**."	424	Case A	2014	[51]
"**Critically ill patients often have low plasma albumin concentrations that alters the protein binding of drugs** and has significant effects on pharmacokinetics. **Given such potential for variability, it is not surprising that we found that one-fifth of patients did not achieve the most conservative PK/PD target and less than 50% of patients achieved what we a priori defined as a preferred PK/PD target.** Furthermore, the variability of unbound concentrations across all antibiotics as well as PK/PD ratios were similarly large. The consequences of insufficient antibiotic exposure may be severe with clear relationships being demonstrated between antibiotic underdosing and the development of antibiotic resistance. This link was initially shown with inappropriately low quinolone exposures, but more recently with other classes of antibiotics including beta-lactams. ICUs are known to harbour multi-drug resistant pathogens and whilst there are many reasons for this, optimised dosing that minimises the evolution of such pathogens should be considered as a method to improve patient and health system outcomes."	14		2014	[52]

"**Furthermore, protein binding is altered in the severely ill, resulting in altered unbound drug concentrations. These alterations in pharmacokinetics and unbound drug concentrations can lead to changes in dosing requirements**."	1324		2014	[53]
"Recently, plasma protein binding (PPB) has been investigated as an additional factor influencing the pharmacokinetics (PK) of antimicrobial agents. Since hypoalbuminemia occurs in approximately 40% of critically ill patients, the potentially negative effects of altered protein binding of antimicrobials may be common. Unbound drug concentrations can differ among patients and underlying disorders, resulting in different responses to therapy or toxicity, as only the unbound drug con- centration exhibits pharmacological activity. **Hypoalbuminemia usually results in higher unbound drug concentrations in plasma. Because the temporary increase in the unbound plasma concentration is reversed by the rapid distribution and elimination of the drug via the liver or the kidneys, this phenomenon is expected to be clinically relevant only for highly protein-bound drugs (PPB above 70%)**. However, for drugs such as voriconazole, with nonlinear pharmacokinetics, the elevated unbound drug concentration in plasma caused by decreased plasma albumin concentrations cannot be instantly metabolized and eliminated. This can be explained by its saturated metabolism and the fact that only 2% of voriconazole is excreted unchanged in urine. **Although voriconazole PPB is only 50%, this saturated metabolism is hypothesized to cause clinically relevant variations in unbound fractions in cases of hypoalbuminemia, potentially resulting in an increased risk for toxic adverse events, even with a total voriconazole trough concentration (VTC) within the reference range of 1 or 2 up to 5.5 mg/liter**."	6782		2014	[33]
"Although voriconazole PPB is documented to be moderate (50%) in patients with normal plasma albumin levels, **hypoalbuminemia can alter voriconazole PPB, probably due to the saturated hepatic metabolism. Increased unbound voriconazole plasma concentrations in patients with profound hypoalbuminemia can possibly cause adverse events, even when total voriconazole plasma concentrations are within the reference range**. Likewise, measured total voriconazole concentrations should be adjusted via the proposed formula, in patients suffering from hypoalbuminemia and showing adverse events potentially related to voriconazole, especially in those with severe hypoalbuminemia."	6787			
"We conclude that **despite physiological plausibility the clinical relevance of hypoalbuminemia for the unbound fraction of vancomycin has not been demonstrated convincingly. This may be due to interactions with other proteins or comedications in clinical samples,** or due to	322	**Wrong sentence:** There is no influence of hypoalbuminemia on unbound/free	2014	[10]

analytical errors in the determination of the unbound fraction."		concentrations and thus no clinical modifications.		
"Linezolid is used for treatment of patients with critical infections caused by Gram-positive bacteria resistant to other antibiotic agents. Critically ill patients often develop hypoalbuminaemia, renal and/or hepatic dysfunction and blood circulatory dysfunction. The effects of hypoalbuminaemia on antibiotic pharmacokinetics are driven by the decrease in the extent of antibiotics bound to albumin. The free fraction of an antimicrobial agent is known to influence the tissue distribution and plasma clearance of the parent compound. **Hypoalbuminaemia can lead to diversity in the pharmacokinetics of free linezolid and protein binding**. In addition, inflammatory proteins induced by critical illness may also affect the free fraction of linezolid. **The pharmacokinetics of free linezolid in critically ill patients has yet to be clarified**."	329		2013	[54]
"There have been few studies on the characteristics of protein binding of linezolid in critically ill patients. Earlier reports demonstrated that serum albumin was the major binding protein for linezolid. The Saline versus Albumin Fluid Evaluation (SAFE) study defined hypoalbuminaemia as serum albumin levels < 25 g/L. The present study demonstrated that the percentage bound declined in hypoalbuminaemic patients. **Hypoalbuminaemia causes an increase in the plasma concentration of free linezolid**."	333		2013	[54]
"**In conclusion, the plasma level of free linezolid and its ratio to MIC were altered in critically ill patients with renal dysfunction and hypoalbuminaemia**. In addition, this study confirmed that linezolid has excellent distribution in peritoneal fluid, pleural fluid and CSF. Two patients failed to achieve adequate efficacy parameters for linezolid, whilst 13 patients showed potential overexposure to linezolid. **These findings suggest that the monitoring of free linezolid is necessary in critically ill patients**."	334	**Ambiguous sentence:** this sentence wrongly suggests to the reader that the unbound/free concentration of linezolid has to be monitored because its value is increased/decreased when hypoalbuminemia		
"To this end, **it remains controversial as to how protein binding changes alter the time course of unbound drug concentration in plasma, and other body compartments, throughout a dosing interval.** Critically, the unbound concentration is of paramount interest as it determines drug efficacy and potential drug toxicity. However, **rational dose adjustment in the presence of altered protein binding is poorly understood and conflicting views exist in the literature as to the impact of changes in protein binding on drug efficacy**."	2		2013	[55]
"From this figure, **it is evident that the pharmacologically active unbound concentration will be affected by the bound drug and the drug distributed into tissue, with both acting as a reservoir for unbound drug in the blood (central compartment).** As drug clearance occurs, a new equilibrium between bound and distributed drug occurs, **which acts to maintain the unbound drug concentration.**	2			

The unbound drug concentration may decrease faster than the new equilibrium can be established if the rate of distribution from the peripheral compartment or the dissociation from protein binding is slower than drug clearance. This would be rare. **The cardinal feature of the above equilibrium is that unbound concentrations are most likely to decrease later in the dosing interval where clearance has reduced the unbound drug and the reservoirs that support its concentrations.**"				
"**For highly protein bound drugs, changes in the fraction bound will have a much larger overall effect on the unbound concentration.**"	5			
"**For all antibacterials where a larger Vd is likely because of changes in protein binding, larger initial doses are suggested,** particularly for the first 24–48 h. Such higher doses are considered especially necessary in the critically ill given the other pathophysiological changes that occur in these patients and the associated iatrogenic interventions [...] **For concentration-dependent antibacterials such as daptomycin, higher dosing, rather than more frequent dosing, would be appropriate.**"	7	**Wrong sentence:** Those conclusions are given in terms of total concentration and do not consider the unbound/free one.	2013	[55]
"**Hypoalbuminemia is a common finding in critically ill patients and causes a higher free drug concentration**, increased clearance and a greater volume of distribution for moderate to highly protein-bound drugs."	164		2013	[35]
"The mean free drug fraction (16+4.5%) was higher in our critically ill patients with acute renal failure than in healthy volunteers (fraction unbound: 4%–10%). The higher free fraction is consistent with the results for the CVVHD patients of Vilay et al. 13 (17.5+5%), and has also been described in patients with end-stage renal disease and haemodialysis. **Since the unbound substance is relevant for exertion of the drug's effect, a higher free fraction is beneficial in critically ill patients, as it might be associated with sufficient free daptomycin concentrations despite lower Cmax. However, caution is advisable in patients with profound hypoalbuminemia; they might reach lower Cmax levels but have unpredictable high free fractions.** Median sieving coefficient and free fraction seemed to correlate in most patients."	981	**Ambiguous sentence:** This sentence suggests that free/unbound concentration is driven by unbound fraction.	2012	[56]
"Outcomes of antibacterial therapy are heavily dependent on achieving therapeutic concentrations of unbound antibacterial at the target site of infection. Effectiveness of empiric dosing is contingent on assumptions that the actual unbound concentrations and antibacterial pharmacokinetics are consistent with those from dose-finding studies. **There are emerging data that demonstrate significant pharmacokinetic changes for the total fraction of highly**	108	**Ambiguous sentence:** Even if the volume of distribution of the unbound/free form is increased due to fluid extravasation, this phenomenon has no consequence on the antimicrobial effect	2011	[57]

bound antibacterials in patients with hypoalbuminaemia. In the presence of increased CL, the increased Vd appears to still cause a significant prolongation of t½ of the drug, which may be therapeutically advantageous for sustaining antibacterial concentrations throughout the dosing interval for highly susceptible pathogens. However, the decreased concentrations resulting from the increased Vd will affect the likelihood of therapeutic success against pathogens with higher MICs and **therefore higher doses may be required in such situations**. Compounding the sparse level of data available on the serum pharmacokinetics of highly protein bound antibacterials is the absence of data describing unbound serum pharmacokinetics and tissue (the site of many infections) pharmacokinetics for these drugs in patients with hypoalbuminaemia. Further research on this topic is urgently required."		of the drug because the AUC is unchanged while the Cmin is increased.<br><br>+<br>Case E		
"*In vitro* and healthy volunteer studies have shown that flucloxacillin is 95%-97% bound to plasma albumin. **The extent of binding to plasma proteins is highly relevant**, as it is the unbound fraction of drug that produces the pharmacological effect. […] Lower albumin concentrations might alter the extent to which flucloxacillin is bound to this protein and, therefore, lead to variations in the unbound fraction of antibiotic that may consequently affect its pharmacokinetics and **pharmacodynamics**. That effect may be additive to the significant variations in pharmacokinetics observed in critically ill patients due to several factors such as the presence of a systematic inflammatory response syndrome (SIRS), fluid resuscitation or the use of inotropes. **The effects of hypoalbuminaemia on the pharmacokinetics of other highly bound antibiotics have been reported previously and suggest a potential risk for inappropriate antibiotic therapy in patients with hypoalbuminaemia. Studying this concept, *Joynt et al.* observed a substantial decrease in fT > MIC (the unbound (pharmacologically active) concentration is maintained above the MIC during a dosing interval) of total ceftriaxone concentrations in critically ill patients with hypoalbuminaemia that led to failure to attain pharmacokinetic–pharmacodynamic targets**. However, a dearth of data exists providing pharmacokinetic–pharmacodynamic simulations for unbound concentrations of highly protein bound drugs. It follows that where such analyses are conducted on total concentrations without accurate knowledge of the free fraction of antibiotic, misinterpretation of the ability of particular dosing schedules to achieve target antibiotic exposures is highly likely."	1772	Case C	2010	[58]
"The data presented in this study provide support for previous studies on altered pharmacokinetics of highly protein-bound antibiotics in critically ill patients with hypoalbuminaemia. Total flucloxacillin V was significantly	1775	Case E		

**larger in our cohort of patients compared with healthy volunteers and other hospitalized, non-critically ill patients, as shown in Table 2. This increase in V may be related, in part, to an increased unbound fraction of flucloxacillin resulting from low plasma albumin levels**. It follows that because unbound drug is the fraction available for distribution and clearance, **the higher fraction of unbound flucloxacillin will be able to distribute to peripheral tissues to a greater extent and explain the larger V**. **Interestingly, we did not observe any significant variation between total flucloxacillin CL in hypoalbuminaemic critically ill patients compared with previous data from healthy volunteers. This is despite data from other antibiotics that suggest that a higher unbound fraction will lead to increased CL because only the unbound molecule is glomerularly filtered**. We have attributed this curious finding to the multiple excretion pathways of flucloxacillin. Flucloxacillin is eliminated by renal (glomerular filtration and tubular secretion, 40% recovery in urine) and non-renal mechanisms (where hepatic metabolism accounts for 30%–40% of total CL). Therefore**, an increase in the glomerular filtration rate resulting from a higher unbound fraction** of flucloxacillin might not be significant enough to alter the total CL, as observed in these patients. However, clarification of this observation can only be achieved by comparison of unbound concentrations between hypoalbuminaemic critically ill patients and healthy volunteers.”				
“Dosing regimens are based primary on PK profiles from healthy volunteers rather than from critically ill patients **where antibiotic disposition is variable and unpredictable due to alterations in intravascular volume, composition of plasma proteins, and renal and hepatic function**. As a consequence of these variables, variations in PK characteristics have been demonstrated for antibiotics such as ciprofloxacin, vancomycin and imipenem. The current study shows lower Cmax and AUC0–∞ and higher Vss, CLT and CLR for total ertapenem compared with those observed in young healthy volunteers. […] **The Cmax and AUC0–∞ of unbound ertapenem were much higher than those in healthy elderly or young adult volunteers and this could be explained by the low serum albumin concentrations that are indicative of the severity of illness in these patients.**”	435	Case A	2009	[59]

"First, in critically ill patients presenting with hypoalbuminaemia, the unbound fraction of **normally moderately** to highly bound drugs may vary, **and so drug clearance may be increased in these circumstances**. Interestingly, **it has recently been shown that this may be clinically relevant especially for the glycopeptides teicoplanin and vancomycin**. Additionally, drug extraction may be further increased by adsorption to the haemofilter membrane, a process whose extent is expected to be maximal immediately after starting RRT and then to progressively decrease over time until filter exhaustion."	1003	Case E	2007	[60]
"Hypoalbuminemia is common in children admitted to the pediatric intensive care unit. The mean serum albumin concentration in our patients was 2.9 g/dL, similar to that reported by other investigators. *Durward and coworkers* reported a mean serum albumin of 3.0 g/dL in 134 critically ill children admitted to a pediatric intensive care unit. **Hypoalbuminemia is a well-described risk factor for elevated free phenytoin concentrations**. In adults, **serum albumin concentrations** < 3.5 g/dL have previously been shown to **affect phenytoin binding ratios** and to contribute to **phenytoin intoxication**. In our study, albumin concentrations < 2.5 g/dL were found to be particularly problematic."	437		2006	[61]
"It is well-described that **other protein bound medications may alter the free fraction of phenytoin by competing for albumin binding sites**. However, there are few clinical data in children to suggest the extent to which this may occur in clinical practice. As in prior studies involving adults, multiple regression analysis in our study confirmed the impact of valproic acid and cefazolin on phenytoin binding in critically ill children. Backward multiple regression analysis revealed that both **valproic acid and cefazolin independently decrease the extent to which phenytoin is protein bound** ($p < .01$). Although the overall mean free phenytoin fraction in our total population was 0.13, this increased to 0.17 with coadministration of valproic acid and to 0.24 with coadministration of cefazolin. [...] **Critically ill children with severe hypoalbuminemia** (< 2.5 g/dL), **and those taking valproic acid or cefazolin**, exhibit significantly altered phenytoin binding ratios and **their free phenytoin concentrations are particularly elevated**. Considering our findings, we recommend that **free phenytoin concentrations be regularly measured in critically ill children, particularly when adjusting phenytoin doses**."	438	**Ambiguous sentence:** This sentence wrongly suggests to the reader that the unbound/free concentration of phenytoin has to be monitored because its value is increased when hypoalbuminemia.		
"Moreover, because the concentration of binding proteins in extravascular fluids is approximately one third that in plasma, **hypoalbuminemia may result in saturation of binding of highly bound drugs both in plasma and in extracellular fluids and, fina-lly, in modifications of the pharmacologic effects of these drugs.**"	735	**Ambiguous sentence:** This sentence wrongly suggests that hypoalbuminemia influences unbound/free concentration.	2000	[40]

"Because only free drug is considered to be pharmacologically active, **alterations of plasma protein binding may alter a patient's response to pharmaceutical agents if protein binding is restrictive for receptor binding. Such changes appeared to be greater with highly bound agents.** Traditionally, most drug assays monitor total drug concentrations and do not quantitate free drug. When binding modifications occur, total drug concentrations may mislead the clinicians' evaluation of the patient's response."	739			
"**However, the increase free drug concentration in plasma of hypoproteinemia patients may enhance the potential drug toxicity, especially when low therapeutic index drugs are used.** Recently, seizures were reported in a hypoalbuminemic patient receiving phenytoin. Despite a therapeutic serum phenytoin concentration, the free concentration was more than doubled. After lowering the daily phenytoin dose, the patient improved. This report clearly indicates better phenytoin distribution in hypoalbuminemic patients with toxic drug concentrations in tissues. [...] **In conclusion, this study indicates that in plasma, iatrogenic hypoalbuminemia induces greater free ceftriaxone concentration, a cephalosporin that binds strong to serum albumin. The higher free drug concentration observed in these hypoproteinemic patients increases drug distribution and may have greater effectiveness.**"	742	**Wrong sentence:** Hypoalbuminemia leads to an increased total volume of distribution and total clearance but does not have any effect on the unbound/free pharmacokinetics. As a consequence, hypoalbuminemia does not have greater effectiveness.		

References

1. Lodise T.P.; Van Wart S.; Sund Z.M.; Bressler A.M.; Khan A.; Makley A.T.; Hamad Y.; Salata R.A.; Silveira F.P.; Sims M.D.; Kabchi B.A.; Saad M.A.; Brown C.; Oler R.E. Jr; Fowler V. Jr; Wunderink R.G. Pharmacokinetic and Pharmacodynamic Profiling of Minocycline for Injection following a Single Infusion in Critically Ill Adults in a Phase IV Open-Label Multicenter Study (ACUMIN). *Antimicrob Agents Chemother*. **2021**, 65, e01809-20, doi: 10.1128/AAC.01809-20.
2. Samura M.; Takada K.; Yamamoto R.; Ito H.; Nagumo F.; Uchida M.; Kurata T.; Koshioka S.; Enoki Y.; Taguchi K.; Higashita R.; Kunika N.; Tanikawa K.; Matsumoto K. Population Pharmacokinetic Analysis and Dosing Optimization Based on Unbound Daptomycin Concentration and Cystatin C in Nonobese Elderly Patients with Hypoalbuminemia and Chronic Kidney Disease. *Pharm Res.* **2021**, 38, 1041-1055, doi: 10.1007/s11095-021-03058-0.
3. Leegwater E.; Kraaijenbrink B.V.C.; Moes D.J.A.R.; Purmer I. M.; Wilms E. B. Population pharmacokinetics of ceftriaxone administered as continuous or intermittent infusion in critically ill patients. *J. Antimicrob. Chemother.* **2020**, 75, 6, 1554-1558, doi: 10.1093/jac/dkaa067.
4. Sime F.B.; Byrne C.J.; Parker S.; Stuart J.; Butler J.; Starr T.; Pandey S.; Wallis S.C.; Lipman J.; Roberts J.A. Population pharmacokinetics of total and unbound concentrations of intravenous posaconazole in adult critically ill patients. *Crit. Care*, **2019**, 23, 205, doi: 10.1186/s13054-019-2483-9.
5. Wasmann R.E.; Muilwijk E.W.; Burger D.M.; Verweij P.E.; Knibbe C.A.; Brüggemann R.J. Clinical Pharmacokinetics and Pharmacodynamics of Micafungin. *Clin. Pharmacokinet.* **2018**, 57, 3, 267-286, doi:10.1007/s40262-017-0578-5.
6. Wong G.; Briscoe S.; McWhinney B.; Ally M.; Ungerer J.; Lipman J.; Roberts J.A. Therapeutic drug monitoring of β-lactam antibiotics in the critically ill: direct measurement of unbound drug concentrations to achieve appropriate drug exposures. *J Antimicrob Chemother*. **2018**, 73, 3087-3094, doi: 10.1093/jac/dky314.
7. Schleibinger M.; Steinbach C.L.; Töpper C.; Kratzer A.; Liebchen U.; Kees F.; Salzberger B.; Kees M.G. Protein binding characteristics and pharmacokinetics of ceftriaxone in intensive care unit patients: Unbound ceftriaxone in ICU patients. *Br. J. Clin. Pharmacol.* **2015**, 80, 3, 525-533, doi: 10.1111/bcp.12636.
8. Carlier M.; Stove V.; Wallis S.C.; De Waele J.J.; Verstraete A.G.; Lipman J.; Roberts J.A. Assays for therapeutic drug monitoring of β-lactam antibiotics: A structured review. *Int. J. Antimicrob. Agents*. **2015**, 46, 4, 367-375, doi: 10.1016/j.ijantimicag.2015.06.016.
9. Enokiya T.; Muraki Y.; Iwamoto T; Okuda M. Changes in the pharmacokinetics of teicoplanin in patients with hyperglycaemic hypoalbuminaemia: Impact of albumin glycosylation on the binding of teicoplanin to albumin. *Int J Antimicrob Agents*. **2015**, 46, 164-8, doi: 10.1016/j.ijantimicag.2015.03.010.
10. Kees M.G.; Wicha S.G.; Seefeld A.; Kees F.; Kloft C. Unbound fraction of vancomycin in intensive care unit patients. *J Clin Pharmacol*. **2014**, 54, 318-23, doi: 10.1002/jcph.175.
11. Dasgupta A. Usefulness of monitoring free (unbound) concentrations of therapeutic drugs in patient management. *Clin. Chim. Acta*. **2007**, 377, 1-2, 1-13, doi: 10.1016/j.cca.2006.08.026.
12. Wallenburg E.; Ter Heine R.; De Lange D.W.; Van Leeuwen H.; Schouten J.A.; Ten Oever J.; Kolwijck E.; Burger D.M.; Pickkers P.; Gieling E.M.; De Maat M.M; Frenzel T.; Brüggemann R.J. High unbound flucloxacillin fraction in critically ill patients. *J. Antimicrob. Chemother.* **2021**, 76, 12, 3220-3228, doi: 10.1093/jac/dkab314.
13. Aulin L.B.S. De Paepe P.; Dhont E.; De Jaeger A.; Vande Walle J.; Vandenberghe W.; McWhinney B.C.; Ungerer J.P.J.; Van Hasselt J.G.C.; De Cock P.A.J.G. Population Pharmacokinetics of Unbound and Total Teicoplanin in Critically Ill Pediatric Patients. *Clin. Pharmacokinet.* **2021**, 60, 3, 353-363, doi: 10.1007/s40262-020-00945-4.
14. Gijsen M.; Dreesen E.; Van Daele R.; Annaert P.; Debaveye Y.; Wauters J.; Spriet I. Pharmacokinetic/Pharmacodynamic Target Attainment Based on Measured versus Predicted Unbound Ceftriaxone Concentrations in Critically Ill Patients with Pneumonia: An Observational Cohort Study. *Antibiotics* (Basel). **2021**, 11, 10, 557, doi: 10.3390/antibiotics10050557.
15. Asada M.; Nagata M.; Mizuno T.; Uchida T.; Takahashi H.; Makita K.; Arai H.; Kijima S.; Echizen H.; Yasuhara M. Population pharmacokinetics of cefazolin before, during and after cardiopulmonary bypass in adult patients undergoing cardiac surgery. *Eur J Clin Pharmacol*. **2021**, 77, 735-745, doi: 10.1007/s00228-020-03045-1.
16. Ulldemolins M.; Bastida C.; Llauradó-Serra M.; Csajka C.; Rodríguez A.; Badia J.R.; Martín-Loeches I.; Soy D. Once-daily 1 g ceftriaxone optimizes exposure in patients with septic shock and hypoalbuminemia receiving continuous veno-venous hemodiafiltration. *Eur J Clin Pharmacol*. **2021**, 77, 1169-1180, doi: 10.1007/s00228-021-03100-5.
17. Moser S.; Rehm S.; Guertler N.; Hinic V.; Dräger S.; Bassetti S.; Rentsch K.M.; Sendi P.; Osthoff M. Probability of pharmacological target attainment with flucloxacillin in Staphylococcus aureus bloodstream infection: a prospective cohort study of unbound plasma and individual MICs. *J Antimicrob Chemother*. **2021**, 76, 1845-1854. doi: 10.1093/jac/dkab089.
18. Tang Girdwood S.C.; Tang P.H.; Murphy M.E.; Chamberlain A.R.; Benken L.A.; Jones R.L.; Stoneman E.M.; Kaplan J.M.; Vinks A.A. Demonstrating Feasibility of an Opportunistic Sampling Approach for Pharmacokinetic Studies of β-Lactam Antibiotics in Critically Ill Children. *J Clin Pharmacol*. **2021**, 61, 565-573. doi: 10.1002/jcph.1773.
19. Rao Z.; Dang Z.L.; Li B.; Zhu L.; Qin H.Y.; Wu X.A.; Wei Y.H. Determination of Total and Unbound Meropenem, Imipenem/Cilastatin, and Cefoperazone/Sulbactam in Human Plasma: Application for Therapeutic Drug Monitoring in Critically Ill Patients. *Ther. Drug Monit.* **2020**, 42, 4, 578-587, doi: 10.1097/FTD.0000000000000736.
20. Wong G.; Taccone F.; Villois P.; Scheetz M.H.; Rhodes N.J.; Briscoe S.; McWhinney B.; Nunez-Nunez M.; Ungerer J.; Lipman J.; Roberts J.A. β-Lactam pharmacodynamics in Gram-negative bloodstream infections in the critically ill. *J. Antimicrob. Chemother.* **2020**, 75, 2, 429-433, doi: 10.1093/jac/dkz437.

21. Abdulla A.; Dijkstra A.; Hunfeld N.G.M.; Endeman H.; Bahmany S.; Ewoldt T.M.J.; Muller A.E.; Van Gelder T.; Gommers D.; Koch B.C.P. Failure of target attainment of beta-lactam antibiotics in critically ill patients and associated risk factors: a two-center prospective study (EXPAT). *Crit. Care Lond. Engl.* **2020**, 24, 1, 558, doi: 10.1186/s13054-020-03272-z.
22. Grégoire N.; Marchand S.; Ferrandière M.; Lasocki S.; Seguin P.; Vourc'h M.; Barbaz M.; Gaillard T.; Launey Y.; Asehnoune K.; Couet W.; Mimoz O. Population pharmacokinetics of daptomycin in critically ill patients with various degrees of renal impairment. *J. Antimicrob. Chemother.* **2019**, 74, 1, 117-125, doi: 10.1093/jac/dky374.
23. Guilhaumou R. Benaboud S.; Bennis Y.; Dahyot-Fizelier C.; Dailly E.; Gandia P.; Goutelle S.; Lefeuvre S.; Mongardon N.; Roger C.; Scala-Bertola J.; Lemaitre F.; Garnier M. Optimization of the treatment with beta-lactam antibiotics in critically ill patients-guidelines from the French Society of Pharmacology and Therapeutics and the French Society of Anaesthesia and Intensive Care Medicine. *Crit. Care*. **2019**, 23, 104, doi: 10.1186/s13054-019-2378-9.
24. Sime F.B. Lassig-Smith M.; Starr T.; Stuart J.; Pandey S.; Parker S.L.; Wallis S.C.; Lipman J.; Jason A. Roberts J.A. Population Pharmacokinetics of Unbound Ceftolozane and Tazobactam in Critically Ill Patients without Renal Dysfunction. *Antimicrob. Agents Chemother.* **2019**, 63, 10, e01265-19, doi: 10.1128/AAC.01265-19.
25. Stein G.E.; Smith C.L.; Scharmen A.; Kidd J.M.; Cooper C.; Kuti J.; Mitra S.; Nicolau D.P.; Havlichek D.H. Pharmacokinetic and Pharmacodynamic Analysis of Ceftazidime/Avibactam in Critically Ill Patients. *Surg. Infect.* **2019**, 20, 1, 55-61, doi: 10.1089/sur.2018.141.
26. Dhaese S.A.M.; Thooft A.D.J.; Farkas A.; Lipman J.; Verstraete A.G.; Stove V.; Roberts J.A.; De Waele J.J. Early target attainment of continuous infusion piperacillin/tazobactam and meropenem in critically ill patients: A prospective observational study. *J Crit Care*. **2019**, 52, 75-79, doi: 10.1016/j.jcrc.2019.04.013.
27. Alexandre K; Fantin B. Pharmacokinetics and Pharmacodynamics of Temocillin. *Clin. Pharmacokinet.* **2018**, 57, 3, 287-296, doi: 10.1007/s40262-017-0584-7.
28. Zheng C.; Hu X.; Zhao L.; Hu M.; Gao F. Clinical and pharmacological hallmarks of rifapentine's use in diabetes patients with active and latent tuberculosis: do we know enough? *Drug Des Devel. Ther.* **2017**, 11, 11, 2957-2968, doi: 10.2147/DDDT.S146506.
29. De Cock P.A.; Desmet S.; De Jaeger A.; Biarent D.; Dhont E.; Herck I.; Vens D.; Colman S.; Stove V.; Commeyne S.; Vande Walle J.; De Paepe P. Impact of vancomycin protein binding on target attainment in critically ill children: back to the drawing board? *J Antimicrob Chemother*. **2017**, 72, 801-804, doi: 10.1093/jac/dkw495.
30. Tsai D. Stewart P.; Goud R.; Gourley S.; Hewagama S.; Krishnaswamy S.; Wallis S.C.; Lipman J.; Roberts J.A. Total and unbound ceftriaxone pharmacokinetics in critically ill Australian Indigenous patients with severe sepsis. *Int. J. Antimicrob. Agents*. **2016**, 48, 6, 748-752, doi: 10.1016/j.ijantimicag.2016.09.021.
31. Laterre P.F.; Wittebole X.; Van de Velde S.; Muller A.E.; Mouton J.W.; Carryn S.; Tulkens P.M.; Dugernier T. Temocillin (6 g daily) in critically ill patients: continuous infusion versus three times daily administration. *J Antimicrob Chemother*. **2015**, 70, 891-8, doi: 10.1093/jac/dku465.
32. Liebchen U.; Kratzer A.; Wicha S.G.; Kees F.; Kloft C.; Kees M.G. Unbound fraction of ertapenem in intensive care unit patients. *J. Antimicrob. Chemother.* **2014**, 69, 11, 3108-3111, doi: 10.1093/jac/dku226.
33. Vanstraelen K.; Wauters J.; Vercammen I.; De Loor H.; Maertens J.; Lagrou K.; Annaert P.; Spriet I. Impact of hypoalbuminemia on voriconazole pharmacokinetics in critically ill adult patients. *Antimicrob. Agents Chemother.* **2014**, 58, 11, 6782-6789, doi: 10.1128/AAC.03641-14.
34. Wong G.; Briscoe S.; Adnan S.; McWhinney B.; Ungerer J.; Lipman J.; Roberts J.A. Protein Binding of β-Lactam Antibiotics in Critically Ill Patients: Can We Successfully Predict Unbound Concentrations? *Antimicrob. Agents Chemother.* **2013**, 57, 12, 6165-6170, doi: 10.1128/AAC.00951-13.
35. Hayashi Y.; Lipman J.; Udy A.A.; Ng M.; McWhinney B.; Ungerer J.; Lust K.; Roberts J.A. β-Lactam therapeutic drug monitoring in the critically ill: optimising drug exposure in patients with fluctuating renal function and hypoalbuminaemia. *Int J Antimicrob Agents*. **2013**, 41, 162-6, doi: 10.1016/j.ijantimicag.2012.10.002.
36. Vilay A.M.; Grio M.; Depestel D.D.; Sowinski K.M.; Gao L.; Heung M.; Salama N.N.; Mueller B.A. Daptomycin pharmacokinetics in critically ill patients receiving continuous venovenous hemodialysis. *Crit. Care Med.* **2011**, 39, 1, 19-25, doi: 10.1097/CCM.0b013e3181fa36fb.
37. Benko R.; Matuz M.; Doro P.; Peto Z.; Molnar A.; Hajdu E.; Nagy E.; Gardi J.; Soos G. Pharmacokinetics and pharmacodynamics of levofloxacin in critically ill patients with ventilator-associated pneumonia. *Int J Antimicrob Agents*. **2007**, 30, 162-168, doi: 10.1016/j.ijantimicag.2007.03.016.
38. Burkhardt O.; Kumar V.; Katterwe D.; Majcher-Peszynska J.; Drewelow B.; Derendorf H.; Welte T. Ertapenem in critically ill patients with early-onset ventilator-associated pneumonia: pharmacokinetics with special consideration of free-drug concentration. *J. Antimicrob. Chemother.* **2006**, 59, 2, 277-284, doi: 10.1093/jac/dkl485.
39. Mimoz O.; Rolland D.; Adoun M.; Marchand S.; Breilh D.; Brumpt I.; Debaene B.; Couet W. Steady-state trough serum and epithelial lining fluid concentrations of teicoplanin 12 mg/kg per day in patients with ventilator-associated pneumonia. *Intensive Care Med*. **2006**, 32, 775-9, doi: 10.1007/s00134-006-0136-3.
40. Mimoz O.; Soreda S.; Padoin C.; Tod M.; Petitjean O.; Benhamou D. Ceftriaxone pharmacokinetics during iatrogenic hydroxyethyl starch-induced hypoalbuminemia: a model to explore the effects of decreased protein binding capacity on highly bound drugs. *Anesthesiology*. **2000**, 93, 735-43, doi: 10.1097/00000542-200009000-00023.
41. Ochs H.R.; Greenblatt D.J.; Woo E. Clinical pharmacokinetics of quinidine. *Clin. Pharmacokinet.* **1980**, 5, 2, 150-168, doi: 10.2165/00003088-198005020-00003.

42. Booke H.; Frey O.R.; Röhr A.C.; Chiriac U.; Zacharowski K.; Holubec T.; Adam E.H. Excessive unbound cefazolin concentrations in critically ill patients receiving veno-arterial extracorporeal membrane oxygenation (vaECMO): an observational study. *Sci Rep.* **2021**, 20, 11, 16981, doi: 10.1038/s41598-021-96654-4.
43. Gatti M.; Bartoletti M.; Cojutti P.G.; Gaibani P.; Conti M.; Giannella M.; Viale P.; Pea F. A descriptive case series of pharmacokinetic/pharmacodynamic target attainment and microbiological outcome in critically ill patients with documented severe extensively drug-resistant Acinetobacter baumannii bloodstream infection and/or ventilator-associated pneumonia treated with cefiderocol. *J Glob Antimicrob Resist.* **2021**, 27, 294-298, doi: 10.1016/j.jgar.2021.10.014.
44. Jager N.G.L.; van Hest R.M.; Xie J.; Wong G.; Ulldemolins M.; Brüggemann R.J.M.; Lipman J., Roberts J.A. Optimization of flucloxacillin dosing regimens in critically ill patients using population pharmacokinetic modelling of total and unbound concentrations. *J. Antimicrob. Chemother*. **2020**, 75, 9, 2641-2649, doi: 10.1093/jac/dkaa187.
45. Van Raaij J.J.; Mabelis N.J.D.; Shudofsky K.N.; Meenks S.D.; Le Noble J.L.M.L.; Janssen P.K.C. Quantification of total and unbound cefuroxime in plasma by ultra-performance liquid chromatography tandem mass spectrometry in a cohort of critically ill patients with hypoalbuminemia and renal failure », *J. Clin. Lab. Anal.* **2020**, 34, 3, e23100, doi: 10.1002/jcla.23100.
46. Kalaria S.N.; Gopalakrishnan M.; Heil E.L. A Population Pharmacokinetics and Pharmacodynamic Approach To Optimize Tazobactam Activity in Critically Ill Patients. *Antimicrob Agents Chemother*. **2020**, 64, e02093-19, doi: 10.1128/AAC.02093-19.
47. Wilkes S.; van Berlo I.; Ten Oever J.; Jansman F.; Ter Heine R. Population pharmacokinetic modeling of total and unbound flucloxacillin in non-critically ill patients to devise a rational continuous dosing regimen. *Int J Antimicrob Agents*. **2019**, 53, 310-317, doi: 10.1016/j.ijantimicag.2018.11.018.
48. Osthoff M.; Siegemund M.; Balestra G.; Abdul-Aziz M.H.; Roberts J.A. Prolonged administration of β-lactam antibiotics – a comprehensive review and critical appraisal. *Swiss Med. Wkly.* **2016**, 41, doi: 10.4414/smw.2016.14368.
49. Brink A.J.; Richards G.A.; Lautenbach E.E.G.; Rapeport N.; Schillack V.; Van Niekerk L.; Lipman J.; Roberts J.A. Albumin concentration significantly impacts on free teicoplanin plasma concentrations in non-critically ill patients with chronic bone sepsis. *Int. J. Antimicrob. Agents*. **2015**, 45, 6, 647-651, doi: 10.1016/j.ijantimicag.2015.01.015.
50. Roberts J.A.; Udy A.A.; Jarrett P.; Wallis S.C.; Hope W.W.; Sharma R.; Kirkpatrick C.M.; Kruger P.S.; Roberts M.S.; Lipman J. Plasma and target-site subcutaneous tissue population pharmacokinetics and dosing simulations of cefazolin in post-trauma critically ill patients. *J Antimicrob Chemother*. **2015**, 70, 1495-502, doi: 10.1093/jac/dku564.
51. Roberts J.A.; Stove V.; De Waele J.J.; Sipinkoski B.; McWhinney B.; Ungerer P.J.P.; Akova M.; Bassetti M.; Dimopoulos G.; Kaukonen K.M.; Koulenti D.; Martin C.; Montravers P.; Rello J.; Rhodes A.; Starr T.; Wallis S.C.; Lipman J. DALI Study Authors. Variability in protein binding of teicoplanin and achievement of therapeutic drug monitoring targets in critically ill patients: lessons from the DALI Study. *Int. J. Antimicrob. Agents*. **2014**, 43, 5, 423-430, doi: 10.1016/j.ijantimicag.2014.01.023.
52. Roberts J.A.; Paul S.K.; Akova M.; Bassetti M.; De Waele J.J.; Dimopoulos G.; Kaukonen K.M.; Koulenti D.; Martin C.; Montravers P.; Rello J.; Rhodes A.; Starr T.; Wallis S.C.; Lipman J. DALI Study. DALI: defining antibiotic levels in intensive care unit patients: are current β-lactam antibiotic doses sufficient for critically ill patients? *Clin. Infect. Dis. Off. Publ. Infect. Dis. Soc. Am.* **2014**, 58, 8, 1072-1083, doi: 10.1093/cid/ciu027.
53. Eyler R.F.; Vilay A.M.; Nader A.M.; Heung M.; Pleva M.; Sowinski K.M.; DePestel D.D.; Sörgel F.; Kinzig M.; Mueller B.A. Pharmacokinetics of ertapenem in critically ill patients receiving continuous venovenous hemodialysis or hemodiafiltration. *Antimicrob. Agents Chemother.* **2014**, 58, 3, 1320-1326, doi: 10.1128/AAC.02090-12.
54. Yagi T.; Naito T.; Doi M.; Nagura O.; Yamada T.; Maekawa M.; Sato S.; Kawakami J. Plasma exposure of free linezolid and its ratio to minimum inhibitory concentration varies in critically ill patients. *Int. J. Antimicrob. Agents*. **2013**, 42, 4, 329-334, doi: 10.1016/j.ijantimicag.2013.06.015.
55. Roberts J.A.; Pea F.; Lipman J. The clinical relevance of plasma protein binding changes. *Clin Pharmacokinet*. **2013**, 52, 1-8, doi: 10.1007/s40262-012-0018-5
56. Wenisch J.M.; Meyer B.; Fuhrmann V.; Saria K.; Zuba C.; Dittrich P.; Thalhammer F. Multiple-dose pharmacokinetics of daptomycin during continuous venovenous haemodiafiltration. *J Antimicrob Chemother*. **2012**, 67, 977-83, doi: 10.1093/jac/dkr551.
57. Ulldemolins M.; Roberts J.A.; Rello J.; Paterson D.L.; Lipman J. The effects of hypoalbuminaemia on optimizing antibacterial dosing in critically ill patients. *Clin. Pharmacokinet.* **2011**, 50, 2, 99-110, doi: 10.2165/11539220-000000000-00000.
58. Ulldemolins M.; Roberts J.A.; Wallis S.C.; Rello J.; Lipman J. Flucloxacillin dosing in critically ill patients with hypoalbuminaemia: special emphasis on unbound pharmacokinetic. *J. Antimicrob. Chemother.* **2010**, 65, 8, 1771-1778, doi: 10.1093/jac/dkq184.
59. Brink A.J.; Richards G.A.; Schillack V.; Kiem S.; Schentag J. Pharmacokinetics of once-daily dosing of ertapenem in critically ill patients with severe sepsis. *Int J Antimicrob Agents*. **2009**, 33, 432-6. doi: 10.1016/j.ijantimicag.2008.10.005.
60. Pea F.; Viale P.; Pavan F.; Furlanut M. Pharmacokinetic Considerations for Antimicrobial Therapy in Patients Receiving Renal Replacement Therapy. *Clin. Pharmacokinet.* **2007**, 46, 12, 997-1038, doi: 10.2165/00003088-200746120-00003.
61. Wolf G.K.; McClain C.D.; Zurakowski D.; Dodson B.; McManus M.L., Total phenytoin concentrations do not accurately predict free phenytoin concentrations in critically ill children. *Pediatr. Crit. Care Med.* **2006**, 7, 5, 434-439, doi: 10.1097/01.PCC.0000235252.43921.DE.
